# Therapeutic Apheresis Using a β2-Microglobulin Removal Column Reduces Circulating Tumor Cell Count

**DOI:** 10.3390/jpm14060640

**Published:** 2024-06-15

**Authors:** Yasuo Komura, Shintarou Kimura, Ayana Takaura, Yumi Hirasawa, Katsunori Segawa, Hiromi Muranishi, Osamu Imataki, Yoshihisa Kumayama, Koichiro Homma

**Affiliations:** 1 Rinku Medical Clinic, 2F Medical Rinku Port, 3-41 Rinku Ouraiminami, Osaka 598-0047, Japan; ykomura@rinku-medical-clinic.com (Y.K.); takaura@rinku-medical-clinic.com (A.T.); segawa@create-consulting.jp (K.S.); muranishi@kyoto.krg.or.jp (H.M.); 2 StateArt Inc., 2-9-12 Horidome-cho, Nihonbashi, Chuo-ku, Tokyo 103-0012, Japan; s.kimura@stateart.co.jp (S.K.); y.hirasawa@stateart.co.jp (Y.H.); 3 Faculty of Medicine, Kagawa University, 1750-1 Ikenobe, Miki-cho, Kita-gun, Kagawa 761-0793, Japan; imataki.osamu@kagawa-u.ac.jp; 4 Osaka ISEN College of Medical Care & Welfare, Osaka 531-0076, Japan; homma888@keio.jp; 5 Department of Emergency and Critical Care Medicine, Keio University School of Medicine, 35 Shinanomachi, Shinjuku-ku, Tokyo 160-8582, Japan

**Keywords:** β2-microglobulin, circulating tumor cell, metastasis, recurrence, therapeutic apheresis

## Abstract

An elevated serum β2-microglobulin (β2M) level is indicative of impaired glomerular filtration and prerenal diseases, such as malignant tumors, autoimmune disorders, and liver diseases. An elevated serum β2M level has been shown to promote metastasis via the induction of epithelial–mesenchymal transition (EMT) in cancer cells. However, the therapeutic potential of targeting β2M remains unclear. Here, we aimed to investigate the efficacy of Filtor, a small polymethyl methacrylate fiber-based β2M removal column, in reducing the β2M level and suppressing cancer cell-induced EMT and metastasis. We assessed the effects of Filtor on the changes in metastasis based on the number of circulating tumor cells (CTCs), which reflects the post-EMT cancer cell population. We performed therapeutic apheresis using Filtor on a male patient with sinonasal neuroendocrine carcinoma, a female patient with a history of colorectal cancer, and another female patient with a history of pancreatic ductal adenocarcinoma. Significantly low serum β2M levels and CTC counts were observed immediately and 4 weeks after treatment compared with those in the pretreatment phase. Moreover, the CTC count immediately after therapeutic intervention was markedly reduced, likely because Filtor had trapped CTCs directly. These findings suggest that therapeutic apheresis with Filtor can prevent cancer metastasis and recurrence by directly removing CTCs.

## 1. Introduction

Understanding the mechanisms underlying cancer cell metastasis and developing effective inhibitory strategies are critical for addressing the challenges of cancer management globally. β2-icroglobulin (β2M), a non-sugar low-molecular-weight protein (11,800 Da) comprising 99 amino acid residues [1], is widely distributed on the plasma membrane surface of all nucleated cells. β2M is noncovalently bound to H chains, in the form of HLA antigen class I (HLA-I) L chains, without being anchored to the surface of the plasma membrane, allowing for dissociation and equilibrium-based exchange with soluble β2M circulating in the extracellular fluid [2]. In disease states, such as infections and cancers, enhanced antigen presentation-induced increases in HLA-I expression promote the dissociation of β2M from HLA-I, leading to elevated serum β2M levels [3]. β2M is largely absorbed by the renal tubules because its low molecular weight allows it to easily pass through the renal glomerular basement membrane. β2M released from the cell surface passes freely through the glomeruli, and 99.9% of it is reabsorbed by the proximal tubules and degraded into amino acids, resulting in only trace amounts being detected in the urine of healthy individuals [3,4]. The half-life of β2M in blood is 2.5 h [4]. β2M reabsorption can be impaired by a decline in renal function and glomerular filtration [3,4], thereby increasing the levels of β2M that are eliminated via urine [3,4]. Therefore, urinary β2M is considered an important marker of tubular damage, especially proximal tubular damage [3,4]. An increased abundance of β2M in lymphocytes and monocytes leads to high serum β2M levels in lymphoid tumors, such as multiple myeloma and autoimmune diseases, indicating that β2M plays an important role in immune response [5]. Moreover, recent studies have demonstrated that serum β2M facilitates the progression of multiple solid tumors, including lung, stomach, and colon cancers, as well as that of blood cancer [5,6]. Therefore, we hypothesized that the removal of circulating β2M inhibits cancer growth and metastasis. 

β2M has been implicated in tumor metastasis via the induction of epithelial–mesenchymal transition (EMT)—a process by which epithelial cancer cells acquire mesenchymal, stemness, and metastatic features [7,8]. Most invasive epithelial cancer cells invade blood and lymph vessels in the form of circulating tumor cells (CTCs) [7], which undergo EMT, followed by engraftment into surrounding and distant organs during metastasis [9]. Moreover, CTCs have been shown to diffuse into the blood even during early tumor development [10]. The presence of CTCs in the blood is considered an indicator of the presence or development of cancer. Additionally, accumulating evidence suggests that CTCs serve as biomarkers in cancer diagnosis and prognosis, as well as surrogate biomarkers of many solid cancers, particularly breast, prostate, lung, and kidney cancers [11]. Thus, testing for circulating CTCs may not only help in predicting cancer metastasis and prognosis but also enable early diagnosis [12]. The simplicity of CTC-based diagnostic tests, which only require blood samples, makes them less invasive than tissue biopsies and are amenable to continuous monitoring for tumor grading purposes [13].

In line with the above reports, we hypothesized that the removal of β2M would suppress EMT and decrease CTC counts. To test this hypothesis, we aimed to investigate whether the removal of circulating β2M via apheresis can reduce blood CTC counts over time and ameliorate cancer progression. In this study, we tested the efficacy of Filtor, a polymethylmethacrylate (PMMA) membrane, in removing circulating β2M and ameliorating cancer progression by monitoring the CTC count.

## 2. Materials and Methods

### 2.1. Patient Cohort

**Figure 1:**
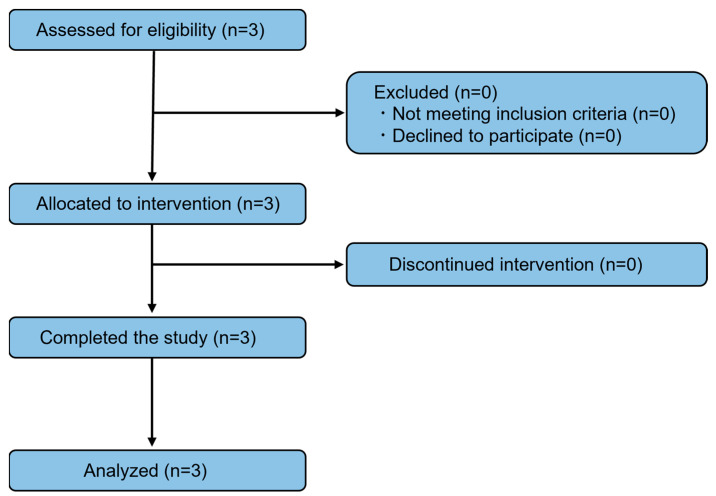
Flow diagram of the clinical trial.

In this clinical trial, patients with cancer or who were at risk of recurrence were included. Patients with severe anemia, chronic renal insufficiency, cirrhosis, deep vein thrombosis, heart failure, and moderate valvular disease, and those undergoing dialysis were excluded from the study. The endpoint of the study was defined as the ability of the patient to undergo apheresis above the circulating blood volume without their quality of life being affected (Figure 1).

### 2.2. Therapeutic Apheresis

We performed therapeutic apheresis, in which the circulating blood of a patient was pumped using a dialysis machine (DCS-27 and NK-Y030PC; Nikkiso Co., Ltd., Tokyo, Japan) fitted with the Filtor membrane (Toray Medical Co., Ltd., Tokyo, Japan) and returned to the body. Apheresis was performed at a filtration flow rate of 100 ± 4 mL/min for 2 h. Approximately one-thirteenth of the patient’s body weight was estimated to correspond to circulating blood volume. Accordingly, we performed blood cleansing at 1.1- to 1.5-times the calculated blood volume, depending on the physical condition of the patient. We collected 10 mL blood from patients using the Blood Access UK Catheter (BA/UK UB-1215-WH; Nipro Co., Osaka, Japan) inserted into the right femoral venipuncture before and immediately after apheresis. Hemostasis was performed via manual compression for 10 min, followed by 1 h of rest before sending the patient home; no rebleeding or hematomas were observed in any of the patients. Blood samples were shipped on ice to Medic Inc. (Shiga, Japan) and Nihon Gene Research Laboratories Inc. (Miyagi, Japan) for the quantification of β2M and CTC, respectively. Nihon Gene Research Laboratories Inc. labelled CTCs that were positive for vimentin and negative for cytokeratin (CK) expression as “Type 1s” if they were single-celled or “Type 1c” if they were clustered, whereas single-celled and clustered CTCs negative for vimentin and positive CK expression were designated as “Type 2s” and “Type 2c,” respectively.

### 2.3. Statistical Analysis

All results are expressed as mean ± standard deviation. The differences between CTC and β2M measurements before and after treatment were analyzed using the one-way analysis of variance (ANOVA) followed by Bonferroni’s post hoc test. The add-in software Statcel4 (v4.0; OMS Publishing, Inc., Tokorozawa, Japan) was used for all statistical analyses and the significance level was set at *p* < 0.05.

## 3. Results

### 3.1. CTC Removal Using Therapeutic Apheresis with Filtor for Sinonasal Neuroendocrine Arcinoma

**Figure 2:**
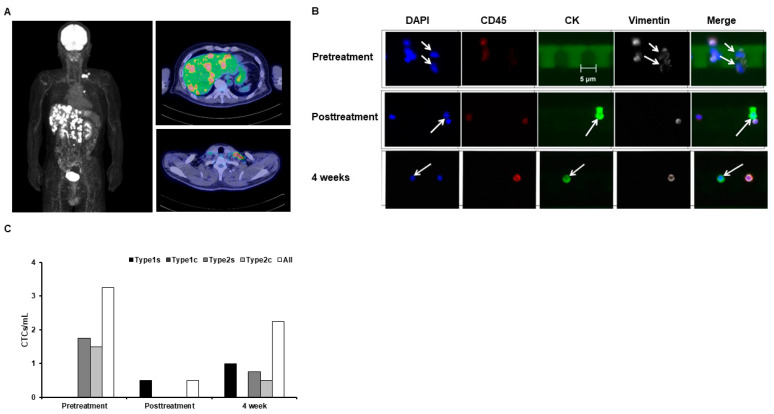
(**A**) Positron emission tomography (PET) images taken before the patient with sinonasal neuroendocrine carcinoma underwent apheresis (October 2023). The frontal image on the left indicates sparsely glowing areas of metastasis. The upper right image indicates multiple hepatic metastases (red). The upper right image of the liver and the lower right image of the left clavicle indicate multiple metastases (red). (**B**) Fluorescence microscopy image of circulating tumor cells (CTCs), where total DAPI indicates cell count, CD45 indicates leukocytes, and cytokeratin (CK) and vimentin indicate Type 1 and 2 CTCs, respectively. (**C**) CTC count per milliliter blood, with “s” indicating single cells and “c” indicating clusters.

The characteristics of the three patients included in this study are shown in Table 1. Our first participant was a 58-year-old man (height: 165 cm, weight: 60 kg) who visited Wakayama Rosai Hospital in 2012 complaining of discomfort in his left nostril and was diagnosed with a stage 2 (T2N0M0) poorly differentiated neuroendocrine carcinoma of the nasal cavity and paranasal sinuses based on pathological diagnosis. Despite receiving systemic anticancer drugs and radiation therapy for the nasal cavity carcinoma, the tumor state worsened, and liver metastases were observed in 2020. In 2023, PET scans revealed liver, intra-abdominal lymph node, and left supraclavicular lymph node metastases (Figure 2A). To prevent further cancer metastasis, we performed therapeutic apheresis for β2M removal at Rinku Medical Clinic. The circulating blood volume of the patient was calculated as 60/13 = 4.615 (4165 mL). Owing to extensive cancer metastasis, we performed therapeutic apheresis at a volume of 6000 mL to ensure blood cleansing. We observed high counts of high-grade Type 2 CTCs before apheresis, consistent with a history of a high incidence of cancer metastasis (Figure 2B,C). Therapeutic apheresis remarkably reduced CTC counts compared to pretreatment levels (Figure 2C). However, the CTC count increased 4 weeks after treatment (Figure 2C).

### 3.2. CTC Removal Using Therapeutic Apheresis with Filtor for Colorectal Cancer

**Figure 3:**
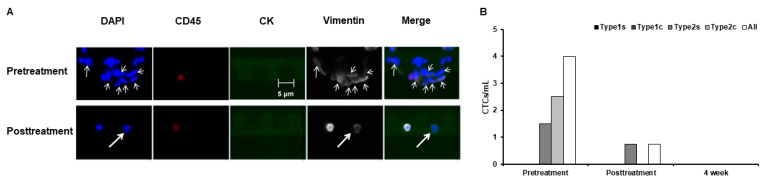
(**A**) Fluorescence microscopy of CTCs acquired from the patient with colorectal cancer, where DAPI indicates total cell count, CD45 indicates leukocytes, and CK and vimentin indicate Type 1 and 2 CTCs, respectively. (**B**) CTC count per mL blood.

Our second participant was a 53-year-old woman (weight: 50 kg) who underwent laparoscopic surgery for stage 1 colorectal cancer at Rinku Medical Clinic in December 2022 and was followed up until October 2023 without any suspicion of metastasis. Based on the wish of the patient, we performed therapeutic apheresis as a measure to prevent cancer recurrence. Her circulating blood volume was 3846 mL; therefore, apheresis was performed at a volume of 4500 mL (~20% more than the assumed circulating blood volume to ensure complete blood cleansing). The CTC count was higher than that expected before apheresis and predominated by high-grade Type 2 CTCs. Type 2 clusters were removed after apheresis (Figure 3A,B), and the total CTC count decreased to undetectable levels after 4 weeks (Figure 3B).

### 3.3. CTC Removal Using Therapeutic Apheresis with Filtor for Pancreatic Ductal Adenocarcinoma

**Figure 4:**
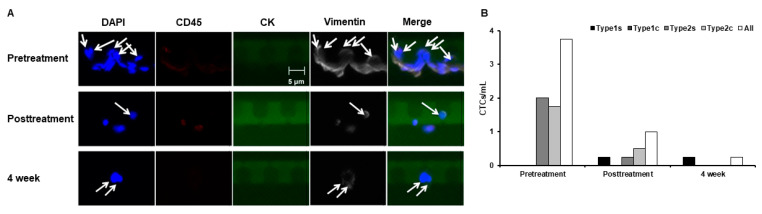
(**A**) Fluorescence microscopy of CTCs acquired from the patient with pancreatic ductal adenocarcinoma, where DAPI indicates total cell count, CD45 indicates leukocytes, and CK and vimentin indicate Type 1 and 2 CTCs, respectively. (**B**) CTC count per mL of blood.

Our final participant was a 69-year-old woman (weight: 52.8 kg) who was diagnosed with pancreatic cancer in July 2022 and underwent a major pancreatectomy followed by systemic anticancer therapy. Although the PET scans did not indicate any affected areas, lymph node sizes tended to increase, prompting the resumption of anticancer treatment in August 2023. On 14 November 2023, the patient underwent apheresis with Filtor at the Rinku Medical Clinic to prevent cancer metastasis. The patient’s circulating blood volume was 4062 mL; accordingly, apheresis was performed at a volume of 4500 mL (~10% more than the assumed circulating blood volume to ensure complete blood cleansing). Highly malignant Type 2 CTCs were detected before apheresis, even though no metastasis was observed (Figure 4A,B). Consistent with the observations of the second participant, we observed a decrease in the CTC count after apheresis, which seemed to persist even after 4 weeks (Figure 4B).

### 3.4. Serum β2M and CTC Counts

**Figure 5:**
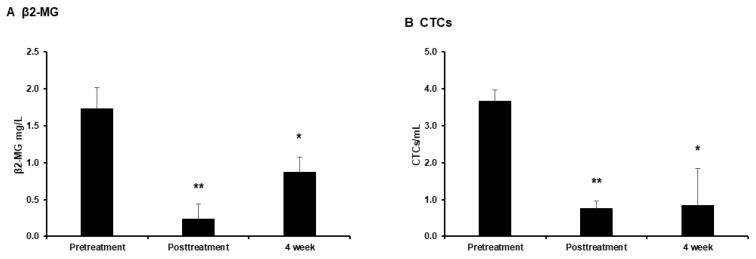
(**A**) Serum β2M levels before, after, and 4 weeks after apheresis. (**B**) CTC counts before, after, and 4 weeks after apheresis. Data are expressed as mean ± standard deviation (* *p* < 0.05, ** *p* < 0.01; ANOVA with Bonferroni’s post hoc test).

Therapeutic apheresis via Filtor significantly reduced the serum β2M levels immediately after treatment compared to pretreatment levels, as per the original specifications (Figure 5A). Although the β2M levels were somewhat restored after 4 weeks, the serum levels remained significantly lower than those at pretreatment (Figure 5A). Similarly, the CTC counts were significantly lower immediately after apheresis compared to those before apheresis (Figure 5B). After 4 weeks, the CTC counts remained significantly lower than those before apheresis, although they were higher than those immediately after apheresis. These changes were similar to those in the serum β2M levels (Figure 5B).

## 4. Discussion

Despite numerous advances in medical technology, the efficacy of cancer treatment is poor. Metastasis to other organs often leads to complications that adversely affect the prognoses and outcomes of the disease [14]. Effective management of metastasis and recurrence, as well as the elucidation of mechanisms underlying cancer cell metastasis and the development of effective inhibitory methods, are essential for improving treatment outcomes. In this study, we demonstrated that therapeutic apheresis utilizing PMMA-based β2M removal columns significantly eliminates not only β2M proteins but also CTCs for at least 4 weeks (Figure 5).

We initially hypothesized that the removal of β2M would inhibit cancer cell EMT and reduce CTC counts. We anticipated that the CTC count would remain unchanged immediately after therapeutic apheresis and decrease only after 4 weeks; however, the CTC counts were considerably low immediately after the intervention compared to those after 4 weeks (Figure 5B). This result indicates that CTCs were directly trapped in the PMMA-based column regardless of the removal of β2M. We hypothesized that this is due to the adhesion of platelets, resulting in platelet–CTC aggregates adhering to the PMMA membrane.

Accumulating evidence shows that hematogenous cancer cell metastasis promotes CTC–platelet interactions and aggregation [15,16,17]. These aggregates protect cancer cells from attack by immune cells in the blood while promoting metastatic nest formation by clogging metastatic organs with microvessels. Podoplanin (PDPN) is an important platelet aggregation-promoting factor expressed on the cell surface of highly metastatic cancer cells [18,19]. PDPN expression is upregulated in squamous cell lung cancer, esophageal cancer, bladder cancer, mesothelioma, glioblastoma, and osteosarcoma, and its expression has been found to positively correlate with metastasis and poor prognosis [19]. The removal of vimentin-positive cells via Filtor in this study may be related to the reported correlation between PDPN and vimentin levels in various cancer types [20,21,22,23]. The negative membrane charge of PMMA prevents cell adhesion, including platelets [24,25,26], whereas platelets recognize it as a foreign object owing to denatured proteins being adsorbed onto its membrane surface [25]. We speculate that this causes platelet-bound CTCs to adhere to the Filtor, resulting in their immediate removal following therapeutic intervention. One concern was that platelets adhering to the PMMA membrane may clog the column via the formation of platelet aggregates, thereby interfering with therapeutic apheresis. However, we did not observe differences in blood flow velocity or a noticeable effect on patient health. This observation may be attributed to Filtor being originally designed to minimize platelet adhesion [26], resulting in significantly reduced CTC cluster levels observed in this study. Collectively, these findings demonstrate that Filtor specifically targeted and captured cancer platelet-bound clusters rather than normal platelets. 

This is a pilot study conducted in a clinic involving only three patients. Therefore, the findings need to be substantiated through further research involving a larger sample. Given the limited number of participants at the clinic, we plan to collaborate with Keio University Hospital to conduct a follow-up study with a large number of participants. Additionally, we have planned to evaluate CTCs by cancer types (e.g., gastric, colorectal, and pancreatic) to account for potential variations in CTC forms and their susceptibility to be captured by Filtor.

## 5. Conclusions

In conclusion, the present study demonstrates that therapeutic apheresis with Filtor effectively removes CTCs, even in patients with highly metastatic cell types. Nevertheless, in addition to the large-scale follow-up investigations, future studies are essential to analyze the morphology, and gene and protein expression of the trapped cells in the column to elucidate the mechanism of CTC entrapment. Moreover, analysis of the CTC-derived genes and proteins detected in Filtor could help undermine the development of novel therapeutics to prevent cancer progression and metastasis. Determining the optimal timing of treatment is also crucial and warrants further exploration, as CTC counts tend to increase 4 weeks after treatment. 

## Figures and Tables

**Table 1:** Clinical characteristics of the three patients included in this study.

Cancer Type	Sinonasal Neuroendocrine Carcinoma	Colorectal Cancer	Ductal Adenocarcinoma
Stage	T2N0M0	T1N0M0	Postoperative follow-up
Age (years)	58	53	69
Sex	Male	Female	Female
Weight (kg)	60	50	52.8
Circulating blood volume (mL)	6000	4500	4500

## Data Availability

The original contributions presented in the study are included in the article; further inquiries can be directed to the corresponding author (KH).
